# The Challenges in the Regulatory Approval of Additive-Manufactured Medical Devices: A Quantitative Survey

**DOI:** 10.1007/s43441-025-00812-z

**Published:** 2025-06-17

**Authors:** Kieran Connole, Olivia McDermott

**Affiliations:** College of Science and Engineering, University of Galway, Galway, Ireland

**Keywords:** Additive manufacturing, 3D printing, Orthopedic devices, Medical devices

## Abstract

In the past decade, additive manufacturing has been applied to mainstream medical devices, particularly in the orthopaedic sector across the hip, knee and shoulder segments for implants and instruments. The research aimed to determine the level of knowledge regarding the regulatory requirements for additive manufactured devices in the Irish Orthopedic medical device sector as well as the challenges faced by orthopedic medical devices manufacturers in interpreting and implementing the current regulatory guidance in the US and the EU. The findings were that there is a lack of knowledge regarding additive manufacturing across the orthopaedic medical device sector and education is required to address this knowledge gap. Furthermore, while the United States has produced specific additive manufacturing guidance, many of the respondents surveyed stated that further clarity is required in this document to remove ambiguity and unclear interpretations of the document. However, in the European Union, there is no support for the use of additive manufacturing as no specific guidance has been provided. This is the first study of its kind on awareness of additive manufacturing regulations. The output of this research is that the Irish orthopaedic medical device sector needs to educate and support its sector members in gaining experience with additive manufacturing in order to ensure that these devices can be commercialised in a timely fashion.

## Introduction

Additive manufacturing or 3D printing to use its more common name is the method of manufacturing whereby a three-dimensional digital rendering of an object is first created, and material is deposited in a layer-by-layer fashion until the finished object is built or completed [18]. Additive Manufacturing printing in the medical device sector has been applied initially to the production of prototype devices and later to custom medical devices. In the past decade there has been an increasing number of medical devices manufactured commercially that are non-customized [3]. An analysis of the additive manufacturing sector outlined that it had grown to a €13.4 billion industry with a 22 per cent annual growth rate, 40 years after the development of the first commercial machines [4].

Additive manufacturing has a wide variety of applications in the aviation and manufacturing sectors [7, 16]. The medical device sector and in particular the orthopedic device sector has been revolutionised by the introduction and advances in this technology. Additive manufacturing can provide anatomical guides for personalised surgery, innovative surgical guides as well as custom-made implants designed to fit a patients anatomy [32].

More recently, additive manufacturing has been applied to mass manufactured orthopedic devices where the patient’s own anatomy is not used to create a specific custom fitting device [3]. This is due to the reduction in the cost of 3D printing equipment as well as the increased availability of machines. 3D printing equipment cycle times have also rapidly decreased making them more competitive against subtractive manufacturing methods [4]. Multinational global orthopedic medical device companies such as Stryker, Zimmer Biomet and DuPuy have all launched implants entirely made from additive manufacturing methods as well as a host of other smaller competitors in the industry.

Since the medical device industry is a highly regulated industry, the safety and efficacy of the AM parts have to be demonstrated and approved through a regulatory framework supported by robust standards [34]. The current regulatory frameworks in the United States and the European Union differ in the level of guidance and detail provided to manufacturer in relation to additive manufactured devices. The US FDA has published a guidance document to support medical device manufacturers and outlines their “Technical Considerations” that must be considered as part of the FDA conformity assessment [15]. On the other hand, the European Union does not have specific guidance for additive manufactured devices and some stakeholders have stated that the EU Commission has been “forgotten” with the introduction of the new medical device regulation [17]. There have been no studies investigating the industry knowledge of AM regulations or of the awareness and views of these regulations as fit for purpose despite arguments that these regulations need to be understood by all stakeholders [19].

This research will also closely examine the challenges for devices manufacturers in gaining regulatory approval for additive manufacturer devices to place their devices on the US and European Union markets. The research questions are as follows:

1. Determine the level of knowledge regarding the regulatory requirements for additive manufactured devices in the Irish Orthopedic medical device sector?
2. Determine the challenges faced by orthopedic medical devices manufacturers in interpreting and implementing the current regulatory guidance in the US and the EU.
3. What can the cross functional teams including regulatory, quality engineering and other functions do to ensure they create a successful additive manufacturing regulatory strategy?

Section "Literature Review" outlines the literature review, while Section "Research Methodology" discuss the methodological approach. Sections "Results and Data Analysis", "Discussion" and "Conclusion" respectively present the results, provide a discussion of the results and conclusion of the study.

## Literature Review

Additive manufacturing or three-dimensional printing is the overarching term for manufacturing technologies that use computer aided design files to fabricate via joining layer by layer of material together to create a three-dimensional object [18]. The key to additive manufacturing success is that the component is fabricated in a thin cross-section from the original computer aided design file by adding one layer at a time. However, the method in which each layer is added can vary due to the technology used to form the bond between each layer and the material being applied.

Additive manufacturing is an advanced manufacturing process that is distinct from conventional manufacturing techniques like casting, forging, and machining [25]. Additive manufacturing instead can be made from powder material whether it be polymer or metal based. An added bonus is that the powder material particularly metals can be recycled such as in powder bed fusion additive manufacturing [18].

### Additive Manufacturing Processes and Techniques in the Medical Device Sector

Additive manufacturing in medical applications was initially used for rapid prototyping as a research and development tool, not for the general manufacture of medical devices [39]. Additive manufacture has also been applied to custom made implants based on a patient’s own anatomy and even cases of devices being printed on site as hospital facilities [40]. However, according to research by the firm from McKinsey, additive manufacturing has become more mainstream across the healthcare sector in the last number of years due to improvements in equipment cycle times [4]. Developments in areas such as orthopedic device implants, prosthetics and surgical devices seem more complex have become more common [20].

Additive manufacturing equipment speeds have improved as well as the wide availability and supplies of metal and polymers required for these machines [35]. According to ASTM/ISO 52900, there are currently five different additive manufacturing process [21] widely applied to medical device manufacturing.

### Regulation of Additive Manufacturing in the United States

The Center for Devices and Radiological Health (CDRH) is the center within the FDA that is responsible for overseeing medical devices in the United States.

There are many different additive manufacturing processes applied to medical device such as stereolithography, fused filament fabrication, polymer jetting or powder bed fusion as well as a variety of materials including titanium, stainless steel and other metals. While additive manufacturing is a new technology, the application in medical device manufacturing does not necessarily necessitate that all devices apply through the Pre-Market Approval (PMA) pathway unless the device design or intended use raises new questions of safety and effectiveness. In fact most AM devices are cleared through the 510 K pathway [14].

Additive manufactured devices themselves do not fall into any special class nor contain any particular product code [15]. The use of additive manufacturing may present additional technical challenges in terms of different manufacturing process flow, biocompatibility or sterilisation methods for the specific device. However, it does not, generally, introduce new questions of safety or effectiveness. From the FDA’s viewpoint the same device that can be manufactured to the same design specifications that is manufactured via conventional methods such as CNC milling and one that has been manufactured using an additive manufacturing methodology would be classified under the same product code regardless of the method of manufacture [6]. Although the FDA have stated that “*in rare cases, additive manufacturing may raise different questions of safety and/or effectiveness*” [9].

For over a decade until 2016, the FDA had been able to regulate additive manufactured devise under existing regulations through the identification of similarities with existing manufacturing technologies and focusing on the key differences between the two in order to complete their evaluation [9]. With the increase in submissions and uncertainty of the impact the safety and effectiveness of the devices under review, the agency took increased interest in the overall area of additive manufacturing.

Subsequently in May 2016, the FDA published a draft guidance document entitled “*Technical Considerations for Additive Manufactured Medical Devices*”. The guidance was finalised in December 2017. The document represents the Agency’s “*initial thinking on technical considerations specific to devices using additive manufacturing, the broad category of manufacturing encompassing 3-dimensional (3D) printing*.” as well as “*recommendations for testing and characterization for devices that include at least one additively manufactured component or additively fabricated step*” [15]. The FDA places the responsibility on the device manufacturer for determining and justification why considerations are applicable or not applicable as the case may be. The key determining factor is the type of additive process being applied such as for example powder bed fusion, stereolithography, fused filament fabrication, or liquid-based extrusion as well as the intended use of the device being submitted for clearance or approval [15].

### Regulation of Additive Manufacturing in the European Union

The new Medical Device Regulation “Regulation (EU) 2017/745 of the European Parliament and of the Council of 5th April 2017 on medical devices, amending Directive 2001/83/EC, Regulation (EC) No 178/2002 and Regulation (EC) No 1223/2009 and repealing Council Directives 90/385/EEC and 93/42/EEC” (referred to here after as “MDR”) was published on April 26th, 2017. The new legislation became applicable after a four-year transition period for medical devices (May 2020) and with it brings a number of changes in how medical devices are regulated across the European Union [12]. So substantial were the changes to the industry, that on 20th March 2023, an amendment to MDR was enacted extending the transitional provisions of the MDR in order to allow more time for the successful transition of medical device manufacturers and Notified Bodies alike [11].

The technical documentation should provide suitable objective evidence to show that the device meets the requirements detailed in Annex I of the MDR 2017/745 General Safety and Performance Requirements (GSPRs) such as the inclusion of reports and detailed evidence of conformity. The GSPR’s can be satisfied with the testing methods which have been based on standards, particularity those which have been harmonized with the medical device regulation. However, at this time only a limited number of standards have been harmonized and none for additive manufacturing [12]. The medical device regulation has introduced an ambiguous concept of “*State of Art*”, where manufacturers can be expected to apply the most up to date standards available to them [31]. One standard viable to manufacturers is ISO/ASTM 52904:2019: *Additive manufacturing—Process characteristics and performance—Practice for metal powder bed fusion process to meet critical applications* has been suggested as a useful guide for the use of Powder Bed Fusion systems [21, 37].

The standard, while not comparable in detail to the FDA’s own guidance document provides information on additional test considerations for additive manufacturing such as surface roughness testing, lattice structure analysis for complex coatings and fatigue testing as well as build placement.

However, a number of stakeholders have publicly complained about the lack of detail or guidance for mass produced additive manufactured devices following the publication of the Medical Device Regulation. Renishaw, a major equipment manufacturer for additive manufacturing machines in the medical device sector has asked “*Is the Medical Device Regulation a Threat to Additive Manufacturing*?” [1]. Furthermore, researchers at the German FH Münster University of Applied Sciences that medical device regulation increased the ambiguity around additive manufacturing more than was there under the medical device directive [5]. Therefore, the current guidance in the European Union is not clear and specific guidance would be welcomed by the industry as well as harmonized standards for additive manufactured devices.

### The Use of Standards to Support Additive Manufacturing Device Approval in the US and EU

Consensus standards provide a consensus approach to certain aspects of the evaluation of device safety and effectiveness, such as testing methods, acceptance criteria, and processes to address areas such as risk management and usability.

Currently there are twenty-two recognized consensus standards related to additive manufacturing recognised by the FDA. Medical device manufacturers by using a harmonised standard for their device can presume conformity against the application medical device directive to the medical device regulation.

However, with the transition to the Medical Device Regulation, all the previously harmonised standards have to be reassessed to the new regulation. A number of standards have been harmonized to the MDR including quality management standards such as EN ISO 13485: 2016 Medical devices—Quality management systems—Requirements for regulatory purposes and EN ISO 14971:2019 Medical devices—Application of risk management to medical devices for the application of risk management to medical devices was harmonised in 2022 [13]. For standards which have not yet been harmonised, the use of these standards is considered to be ‘state of the art’ and their use is encouraged i.e., the most up to date standards or accepted methods. Currently there are only a handful of harmonized standards against the Medical Device Regulation, and none are for additive manufacturing. Therefore, medical device manufacturers must apply non-harmonised standards for additive manufactured devices [34]. There are current two families of additive manufactured standards. The first, the ISO/ASTM 52900 series runs from ISO/ASTM 52900 to ISO/ASTM 52950 which includes processing methods and equipment across the entire additive manufacturing process [36]. Given the introduction of “State of Art” in the European Union, manufacturers can be expected to apply the most up to date standards available to manufacturers [31]. Using this route, manufacturers may be able to generate sufficient detail for regulators to review the safety and effectiveness of their devices but only for specific additive manufacturing methods.

### Conclusion

While additive manufacturing is over 40 years old, it is only in recent years that it is being applied to mainstream medical device manufacturing, particularly in the orthopedic medical device sector. While the FDA have invested in understanding additive manufacturing through internal expertise and through the inclusion of industry representatives via workshops, no such effort has been identified in the European union where the focus of regulators has been on the introduction and role out of the new MDR. In the United States, the FDA guidance on additive manufacturing is detailed and provides a framework for manufactures to bring their devices to market. However, in the European Union, there is no clear framework for medical device manufacturers and instead they must rely on the concept of State of the Art in order to use the best available standards and methods at their disposal. Standards are crucial in providing technical considerations to meet regulatory requirements and minimize the risk. Standard development organizations, including ASTM and ISO, are developing AM medical device-specific standards and which with the establishment of regulatory frameworks can ensure the utilization of AM to its fullest potential.

## Research Methodology

Quantitative research was chosen as the methodology of choice as suitable for the subject matter and objectives outlined. The medical device sector is a large industry in Ireland, the number of medical device professionals employed in the orthopedic sector is insignificant at 8% of the total sector [30]. Furthermore, it is difficult to determine how many professionals within the orthopedic sector are using additive manufacturing technology at this time and what their level of experience is due to employees in some organisation being subject to non-disclosure agreements. The aim of this research is to investigate knowledge of regulations of additive manufacturing in mass manufactured devices as opposed to custom made devices.

A survey was designed and piloted with questions as outlined in Table 1. As the validity and reliability of the data collected, and the response rate achieved depend largely on the design of the questions, the structure of the questionnaire, and the rigour of the pilot testing care was taken to get feedback on the survey [2].

**Table 1:** Research survey questions (non-demographic section)

Question	Response	Literature sources
Can you rate your knowledge and awareness of the current standards and regulations used in additive manufacturing for medical devices?	Familiarity ranking from 1 to 5	[9, 29]
Do you actively keep track and identify new standards for additive manufacturing?	Yes/no	[15, 21, 36]
How familiar are you with the ISO/ASTM 52900 standards for Additive Manufacturing?	Familiarity ranking from 1 to 5	[6]
Do the ISO/ASTM 52900 standards provide sufficient detail for qualifying and validating additive manufacturing processes in the Orthopedic Industry?	Rank from 1 to 5 in terms of detail provided	[20, 21]
Are you familiar with of requirements for additive manufacturing in the EU e.g. regulatory guidance and/or harmonized standards?	Familiarity ranking from 1 to 5	[13, 34]
How would you rate the ease or difficulty in having a new additive manufactured product approved in the EU?	Difficulty ranking from 1 to 5	[5, 23]
Are you familiar with the FDA guidance for Additive Manufacturing, Technical Considerations for Additive Manufactured Medical Devices	Familiarity ranking 1–5	[5, 34]
Does the FDA guidance for Additive Manufacturing, Technical Considerations for Additive Manufactured Medical Devices" provide sufficient detail for qualifying and validating additive manufacturing processes in the Orthopedic Industry?	Agreement ranking 1–5	[6, 36]
Is the FDA guidance for Additive Manufacturing, Technical Considerations for Additive Manufactured Medical Devices" difficult or easy to and apply to additive manufacturing processes?	Agreement rank 1–5	[14, 36]
How would you rate the ease or difficulty in having a new additive manufactured product approved in the US?	Rank 1–5: very difficult: very easy	[6, 29]
What additional detail and/or clarity would you like to see in future regulatory guidance documents from the FDA?	Open comment	[34, 36]
Can you outline the top regulatory and/or technical challenges with additive manufacturing in the Orthopedic medical devices?	Open comment	[23, 24, 29]
What methods can be used/deployed by cross functional teams, which include regulatory, quality, engineering, etc., to ensure a successful regulatory strategy is in place for AM medical devices produced for market in the US and the EU?	Open comment	[24, 34]

The survey was created using SurveyMonkeyTM software in order to generate a questionnaire which accessible and efficient in data collection [26]. The survey was distributed to all participants via email, the email included an explanation of the purpose of the survey, and ethical information [22]. The target audience for the survey was strictly for professionals working with additive manufactured devices. Contacts were identified through LinkedIn and participants were limited to those working in the Irish orthopaedic medical device sector and those in large and small enterprises as well as those in academic institutions.

The survey was disseminated to 63 people, of whom 41 people responded, yielding a response rate of 64% which was deemed satisfactory [10]

## Results and Data Analysis

### Survey Demographics

Ireland is home to fourteen of the top fifteen medical device companies and many of these companies are larger than five hundred employees (IDA 2023). In particular, Ireland has the presence of major orthopaedic multinationals such as Stryker, DePuy and Zimmer, all heavily involved in additive manufacturing, as Ireland's 8th highest exported items are orthopaedic appliances. Regulatory, Engineering, Research and Development (R&D) as well as Quality management professionals from across the Irish orthopedic medical device sector were surveyed on their knowledge of additive manufacturing regulations and standards. They were also surveyed on the challenges faced in bringing an additive medical device to market and also asked what the best method is that cross-functional teams can deployed to ensure a successful regulatory strategy.

In terms of the respondent's organisational size, the majority 85.37% worked in an organization with more than five hundred employees or a Large enterprise (LE). The remainder worked in organisations with between two hundred to five hundred employees (Small & Medium Enterprises). The majority of the respondents were from an Engineering (43.90%) followed by Quality (17.07%), Regulatory Affairs (14.63%), and Research & Development (14.63%) functions, respectively. In terms of experience with Additive Manufacturing, nearly 50% of the respondents, stated that they had 1–5 years’ experience while 22% had 5+ years of experience and 28% has less than one years’ experience with additive manufacturing.

Given that additive manufacturing has only been applied to mainstream manufacturing for a short period of time, it is not surprising that the those working in the Irish orthopedic medical device sector do not have extensive experience in this area.

### Knowledge Levels of and Tracking of Additive Manufacturing Regulations

Only just over 7% of respondents stated that they were extremely familiar with the standards and regulations related to additive manufacturing. Some regulations and standards specifically related to AM were provided to ensure AM regulations were not confused with other standards and regulations. A further 17% stated that they were very familiar while the majority of just under 44% stated they were somewhat familiar. A combined total of approx. 32% stated that they were not so familiar and not at all familiar with the standards and regulations in additive manufacturing, respectively. Given that many of the respondents do not have extensive experience with additive manufacturing, it is not surprising that the majority of the respondents do not have familiarity with the standards and regulations of additive manufacturing.

In relation to the first RQ in this study, the level of knowledge and experience in the general regulations and standards has been determined to be low. Given that this is a new area, further training and education for those in the medical device sector may be required to provide greater experience in additive manufacturing and also enhance their knowledge of the regulations and standards in this sector.

A question related to the tracking and identification of new standards for additive manufacturing was asked. The majority of respondents answered No (75%) that they do not track new standards while 25% of respondents answered Yes. Those that answered Yes were working in the Regulatory Affairs functions and R&D functions primarily. Individuals working in Engineering and Quality support functions related to Operations are generally not as involved in keeping abreast of new standards coming through.

Respondents were further asked as to how familiar they were are you with the ISO/ASTM 52900 standards for Additive Manufacturing in particular. These particular standards are the key family of standards for additive manufacturing for medical devices. No respondents stated that they were extremely familiar with this family of standards. Five (12.20%) respondents stated that they were very familiar, and seventeen (41.46%) respondents stated they were somewhat familiar. However, twelve (29.27%) and seven (17.07%) respondents that they were not so familiar or not at all familiar, respectively.

A further question was on the application of ISO/ASTM 52900 series standards in terms of whether they provided enough detail for qualifying and validating additive manufacturing processes in the Orthopedic Industry.

Only one respondent replied that the standards provided sufficient detail for qualifying and validating additive manufacturing processes in the orthopedic industry while nine (23.08%) answered they provided a lot of detail, nineteen (48.72%) answered that they provided a moderate amount, eight (20.51%) answered that they provided a little and two (5.13%) responded that they provided no detail at all.

### Familiarity with EU Additive Regulations

**Fig. 1:**
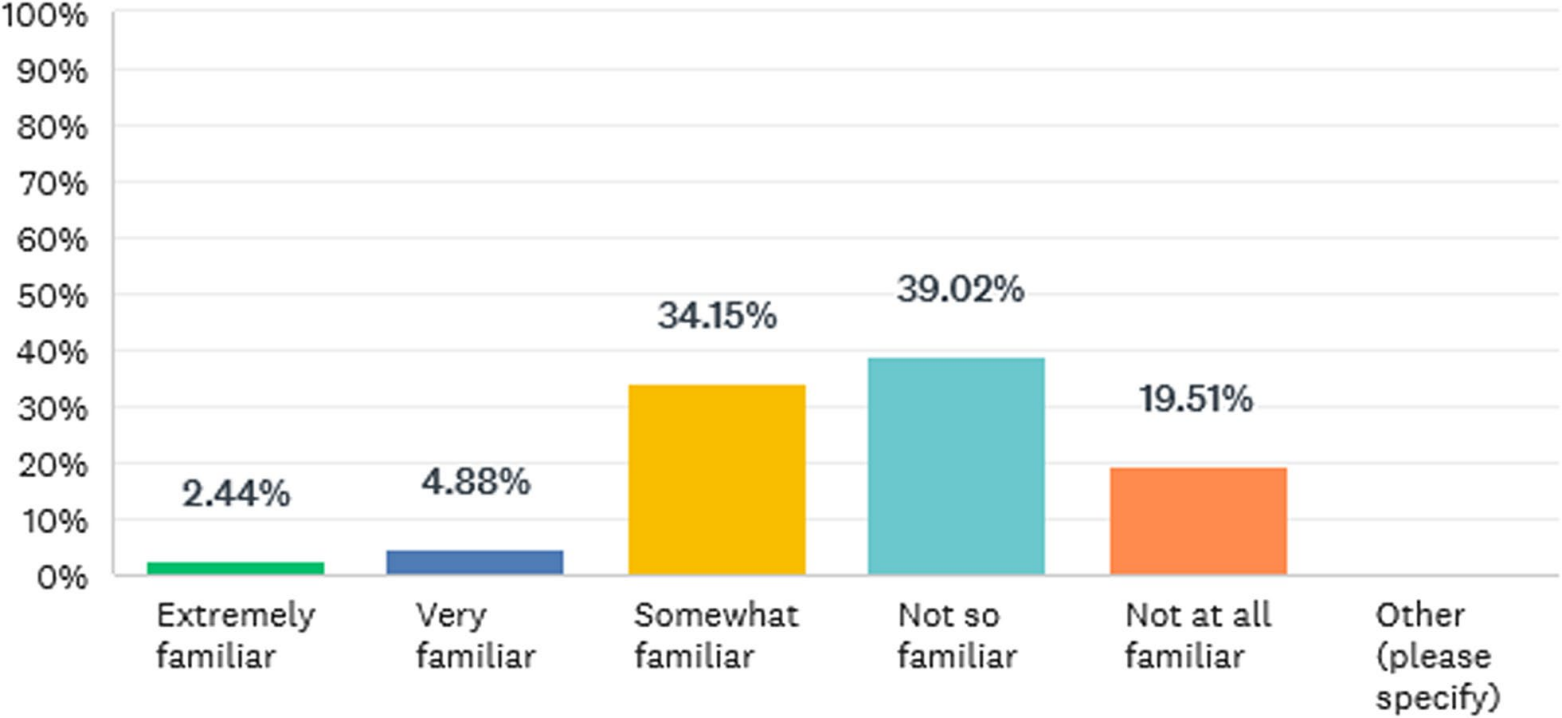
Familiarity with EU AM requirements

The question next asked was: Are you familiar with of any specific requirements for additive manufacturing in the EU e.g. regulatory guidance and/or harmonized standards? All respondents answered this question with no one skipping it. The results are outlined in Fig. 1.

The majority of respondents outlined that they were no, not at all or somewhat familiar with guidance and standards form the European Union.

Survey question ten asked those being surveyed, *How would you rate the ease or difficulty in having a new additive manufactured product approved in the EU*? Not one responder answered very easy or easy. 28.95% of responders answered neither difficult nor easy, 68.42% answered difficult while 2.63% answered very difficult.

Question eleven moved on from regulations in the European Union to the United States. This question asked, *Are you familiar with the FDA guidance document for Additive Manufacturing, Technical Considerations for Additive Manufactured Medical Devices?* A large proportion of those surveyed answered that they were only somewhat familiar with the guidance document with twenty (48.78%). Four (9.76%) and five (12.20%) answered that they were extremely familiar and very familiar respectively. Finally, eight (19.51%) answered that they were not so familiar while four (9.76%) responded they were not familiar at all.

The question was asked: *Does the FDA guidance for Additive Manufacturing, Technical Considerations for Additive Manufactured Medical Devices"* provide sufficient detail for qualifying and validating additive manufacturing processes in the Orthopedic Industry? One person (2.44%) responded that they strongly agreed with this question, twenty (48.78%) answered agree, fifteen (36.59%) answered that they neither agreed nor disagreed with the questions, five (12.20%) answered that they disagreed, and none of those surveyed stated that they strongly agreed.

Question thirteen asked those being surveyed: *How would you rate the ease or difficulty in having a new additive manufactured product approved in the US?* No one responded that the process to have an additive-manufactured device approved in the United States was very easy or easy. Eighteen respondents (45.00%) answered that the process was neither easy nor difficult while twenty-one (52.5%) answered that the process was difficult.

### The Future of Additive Regulations:

An open-ended question was asked about “*What additional detail and/or clarity would you like to see in future regulatory guidance documents from the FDA?”.*

The responses themes from this survey question included more clarity required in general in the FDA guidance document, a more structured approach for qualifying and conducting the process validation for specific technologies used in additive manufacturing as well as more alignment with the international standards from international standard bodies (ISO and ASTM) for additive manufacturing.

The top three themes identified were related to additive process validation with over 40 comments related to this area 40.91% while a further 25 respondents discussed having clearer guidance and about 12 of the responses referring to more information on standards in additive manufacturing.

In relation to the validation responses respondents stated that the FDA’s guidance document. “*Technical Considerations for Additive Manufactured Medical Devices*" required more information on the validation of additive manufacturing as the current guidance is “*ambiguous*”, “*the numerous points in the document that manufacturers should consider is not weighted for importance*”, “*clear expectations are needed for worst case conditions and process controls*”, “*need a more practical set of guidelines* for *process validation*” and “*detailed guidance should be included on process validation specific to the technology in use”. B*ased on the responses, those surveyed feel that the guidance document should be updated include specific detail on validating for specific additive manufacturing techniques. Also, the respondents feel that the document outlines too many consideration that must be taken into account for the validation process with no emphasis on their importance.

Finally, the third theme was around standards used in additive manufacturing. The theme of standards is similar to that of clearer guidance as the respondents feel that there should be *“Standards for Qualifications and Certifications*” and “*more detail regarding requirements of ASTM standards*”. This point is similar to the themes of Validation and Clear Guidance but specific to standards.

Reviewing the three major themes gathered from the aforementioned question respondent’s feel there is a need for further clarity on the application of the guidance documents to specific additive manufacturing techniques and better linkage to the international standards in use. There is also a lack of clarity regrading what elements to consider in validation planning and the technical considerations proposed by the FDA are not stratified by their importance.

The next open unstructured question was “*Can you outline the top regulatory and/or technical challenges with additive manufacturing in the Orthopedic medical devices*?”. As these responses were opened ended questions, there was a wide variety challenges identified by those survey. However, there themes that dominated the responses were particularly around areas like technical understanding of the equipment/processes and the lack of knowledge of additive manufacturing in general.

More than half of the respondents highlighted that they consider the lack of knowledge/experience in additive manufacturing to be a challenge in the manufacturing of AM devices. A further third of respondents considered ambiguous regulatory guidance and interpretations from submission reviewers to be a key challenge to bringing a new additive manufactured devices to market.

Some respondents highlighted that you must “*understand the AM process fully and have identified the critical parameters and effects on the product safety*” as well as “*understanding the inter-system variability and even variability from machine-to-machine for different systems*” which can cause issues with this technology. Other respondents noted that “*getting experienced additive manufacturing workforce”* was a fundamental challenge and another one highlighted was the “*lack of understanding of additive manufacturing*”.

On the interpretation of regulatory guidance, one respondent stated that “*there appears to be uncertainty on both sides of the regulatory interaction, manufacturer and regulator, that leads to some confusion on what would be acceptable for an AM device and how to provide that information, to demonstrate safety and effectiveness*” and another stated that it is “*unclear if all companies in this area are interpreting the guidance in the same way, with potential differences between how regulatory body submission reviewers are policing it*”. Given that the majority of feedback was regarding the FDA regulations, even though there is a guidance document available from the FDA there seems to be differences in interpretation of the requirements between regulatory reviewers and medical device manufacturers.

The last question in relation to the future of additive manufacturing was “*What methods can be used/deployed by cross functional teams, which include regulatory, quality, engineering, *etc*., to ensure a successful regulatory strategy is in place for additive manufactured medical devices produced for market in the US and the EU*?.

One of the main themes for knowledge sharing including main responses were around the “*provision of training so that all departments involved are aware of the standards/regulations, technologies, best practices, *etc.”, “a*ll teams need to be familiar with the additive process from start to finish”, “a AM centre of excellence would be benefici*al”, and “*improve knowledge sharing across the organisation*”. Other participants responded that cross functional teams need to “*review the regulations to ensure clarity*” and have an understanding of “*ASTM/ISO standards or guidance documents within the organization* “. Finally, a sizeable number of respondents responded that “*experience*” with additive manufacturing “*is essential*” to ensure a correct regulatory strategy. Responses included, “*ensure you have appropriate AM experience within the team to help guide the project through the expected outcome*” and have “*experience based on previous AM submissions*” and conduct “*intensive internal and external training needs to be completed by AM companies.*”

## Discussion

### Research Question 1

Research question one was to “*Determine the level of knowledge regarding the regulatory requirements for additive manufactured devices in the Irish Orthopedic medical device sector*?” Following a literature review, there was no data available regarding the level of knowledge of regulatory requirements for additive manufacturing in the Irish orthopedic medical device sector or any other global sector. Several survey questions were used to determine the levels of knowledge regarding additive manufacturing regulatory requirements.

Respondents were asked to rate from one to five their current knowledge and awareness of the current standards and regulations used in additive manufacturing for medical devices. A further set of questions assessed knowledge of the ISO/ASTM 52900 series of standards, European Union regulatory guidance and/or harmonized standards and their familiarity with FDA guidance for Additive Manufacturing, “*Technical Considerations for Additive Manufactured Medical Devices*”. In all of these questions on average, approximately 70–80% of respondents answered that they were only “somewhat” familiar whereas only approximately 20–30% answered that they were “familiar” with the same standards, regulations or guidance documents.

It is clear that the level of knowledge and experience in those respondents regarding the international standards, regulations or guidance documents across the European Union and the United States is low as determined through the survey results. Generally the level of knowledge of regulations and guidance’s can vary depending on how much interaction and training that professionals have with the relevant material especially if newly implemented [23]. Given that this is a new area, further training and education for those in the medical device sector may be required to provide greater experience in additive manufacturing but also enhance their knowledge of the regulations and standards in this sector[38]. One caveat to this research data gathered is that the backbone of the respondents is from the engineering speciality who may or may not be as intimate with standards and regulations as those employed in the quality or regulatory functions. However, these engineers are involved in ensuring the manufacturing process and equipment are validated, supported, and maintained in line with regulations as part of their organisations QMS.

### Research Question 2

Research question two was to “*Determine the challenges faced by orthopedic medical devices manufacturers in interpreting and implementing the current regulatory guidance in the US and EU”* and sought to gather information on the main challenges for additive device manufacturers in the jurisdictions outlined. Information on these challenges were highlighted earlier in the literature review in the United States but particularly in the European Union. While the United States has produced their own guidance document for this area and has recognised standards, in the European Union there are no harmonised standards under the Medical Device regulation or dedicated guidance for medical device manufacturers. One specific survey question asked respondents to highlight their challenges in bringing an additive-manufactured device to market. The results from the survey highlighted two main themes in terms of these challenges, which were that the interpretation of regulatory guidance differed at times between the regulatory authorities and the medical device manufacturers and also that there was a lack of knowledge/experience of additive manufacturing in general. Interpretation of regulatory guidance is a recognised challenge in the industry as Kearney and McDermott [23, 24] have highlighted in their study of clinical evaluation requirements under the new MDR that the guidance documents are not always clear on the regulator's expectations.

Two comments highlighted these challenges from the survey responses. These were “*there appears to be uncertainty on both sides of the regulatory interaction, manufacturer and regulator, that leads to some confusion on what would be acceptable for an AM device and how to provide that information, to demonstrate safety and effectiveness*” and another respondent stated that it is “*unclear if all companies in this area are interpreting the guidance in the same way, with potential differences between how regulatory body submission reviewers are policing it*”. Rafi et al. [34] stated that the available standards only provide some of the required information for the premarket review process for medical device submissions. They gave the example of ASTM F3001, Standard Specification for Additive Manufacturing Titanium-6 Aluminum-4 Vanadium ELI (Extra Low Interstitial) with Powder Bed Fusion needs to have more information to aid determination of final part material properties based on powder reuse or other built conditions.

Given these differences in the interpretation of regulatory guidance it might point more to the United States and the FDA guidance and that there is clarification required in their guidance document. Standards for AM are a need in the industry and the committees of the ASTM F42, the ISO TC261 and CEN TC438 are working collaboratively developing standards applied at global level[27].

### Research Question 3

This research question investigated the themes of “*What can the cross functional teams including regulatory, quality engineering and more do to ensure they create a successful regulatory strategy*?”. No data was available on additive manufacturing device strategy in the literature, but this could be due to a number of issues such as confidentially within additive manufacturing medical device manufacturers and equipment manufacturers and the fact that academic publications have not yet caught up with industry challenges. Typically Medtech and Pharma industries are not inclined to share process information[28] The literature did highlight a number of academic groups completing technical design and manufacturing of the AM devices but did not speak to regulatory processes for a specific device. The survey data yielded a number of responses on this area. A sizeable number of respondents (almost one third) identified that knowledge and knowledge sharing was a key area that should be focused on by cross functional team brings a new device to market. One of the main themes for knowledge sharing including responses as mentioned previously around the provision of “*training so that all departments involved are aware of the standards/regulations, technologies, best practices, *etc.”, and “*All teams need to be familiar with the additive process from start to finish”,* and “*knowledge sharing across the organisation*”. Other participants responded that cross functional teams need to “*review of the regulations to ensure clarity*” and have an understanding of “*ASTM/ISO standards or guidance documents within the organization”.* Finally, several respondents discussed the importance of having AM experience be it in engineering, manufacturing, regulatory or other functions as essential to ensure a correct regulatory strategy. Reponses included “previous AM *project experience”,* have “*experience based on previous submissions*” and have “*extensive AM training*”. Lack of skilled personnel across the AM supply chain was highlighted as the main challenge in AM adoption in the Medtech sector [33]

In summary, material selection and validation, quality control and consistency, regulatory approval processes, post-marketing surveillance, advancement in regulatory frameworks, standardization and certification are industry-wide challenges. Similarly Chowdhury et al. [8] found material characterisation, characteristics of raw materials and costs of materials a challenge for the industry.

It is clear that the Irish orthopedic medical device sector needs to increase the level of knowledge both generally around additive manufacturing and within cross functional teams but also create specialists and internal experts with the knowledge to bring these devices to market. However, the current published standards are not enough to address the existing gaps and these standards only provide some of the information required information for medical device submissions. Based on powder reuse or other built conditions.

Theoretical and managerial implications of this research:

This research has implications for industry practice as it provides insights into getting to market with clarity and outlines the gaps in current regulation as well as the importance of awareness of regulatory timelines and knowledge of standards and regulations. Regulatory professionals can use this study to understand the AM regulatory landscape and its challenges in order to improve its submissions. From a theoretical implications view point this study can aid policy development and practice as well as add to the literature in relation to AM regulatory requirements.

## Conclusion

The study discussed the level of knowledge regarding the regulatory requirements for additive manufactured devices in the Orthopedic medical device sector as well as the challenges faced by orthopedic medical devices manufacturers in interpreting and implementing the current regulatory guidance in the US and the EU; and the role of the cross functional teams and stakeholders in ensuring that they create a successful additive manufacturing regulatory strategy. The level of regulatory guidance in the European Union is poor, and specific guidance is needed, which will eventually impact innovation in the common market. The FDA guidance document, while generally well received and praised, needs further clarification, as do the international standards. While additive manufacturing is a rapidly changing field and one that is full of potential, it is clear that the Irish orthopedic medical device sector must increase the level of knowledge across a number of functions.

Additive manufacturing is a new technology which brings benefits such as the ability to build complex device designs but also presents new hurdles such as ambiguity around the regulation of this technology in different justifications.

This study is novel in that it is the first of its type both in Europe and globally specifically involving orthopaedic additive manufacturers. Its value lies in the sharing of challenges and stakeholder regulatory requirements that can help inform future regulatory policy.

Future research in this area could expand the study with more detailed qualitative studies and case studies with the orthopaedic industry. A limitation of this study was that a number of medical device manufacturers and their staff could not or would not take part in this research project because of confidentiality reasons. Another limitation of the study was that it took place only in Ireland as a single case example of IRS orthopaedic multinationals; however, as Ireland has a high level of orthopaedic exports and is part of Europe, the results can be generalized to other European orthopaedic manufacturers working from the same EU regulations. Additional data gathered via the aforementioned studies would provide a wide overview of the challenges of bringing an additive-manufactured device to market in this important area.

## Data Availability

No datasets were generated or analysed during the current study.
